# Predator-Prey Interactions between Shell-Boring Beetle Larvae and Rock-Dwelling Land Snails

**DOI:** 10.1371/journal.pone.0100366

**Published:** 2014-06-25

**Authors:** Els Baalbergen, Renate Helwerda, Rense Schelfhorst, Ruth F. Castillo Cajas, Coline H. M. van Moorsel, Robin Kundrata, Francisco W. Welter-Schultes, Sinos Giokas, Menno Schilthuizen

**Affiliations:** 1 Focus Group Character Evolution, Naturalis Biodiversity Center, Leiden, The Netherlands; 2 Centre for Ecological and Evolutionary Studies, Rijksuniversiteit Groningen, Groningen, The Netherlands; 3 Department of Animal Ecology and Tropical Biology, University of Würzburg, Würzburg, Germany; 4 St Antonius Hospital, Nieuwegein, The Netherlands; 5 Institute Biology Leiden, Leiden University, Leiden, The Netherlands; 6 Department of Zoology, Palacky University, Olomouc, Czech Republic; 7 Zoologisches Institut der Universität, University of Göttingen, Göttingen, Germany; 8 Department of Biology, University of Patras, Rio (Patras), Greece; Universidade de São Paulo, Faculdade de Filosofia Ciências e Letras de Ribeirão Preto, Brazil

## Abstract

*Drilus* beetle larvae (Coleoptera: Elateridae) are specialized predators of land snails. Here, we describe various aspects of the predator-prey interactions between multiple *Drilus* species attacking multiple *Albinaria* (Gastropoda: Clausiliidae) species in Greece. We observe that *Drilus* species may be facultative or obligate *Albinaria*-specialists. We map geographically varying predation rates in Crete, where on average 24% of empty shells carry fatal *Drilus* bore holes. We also provide first-hand observations and video-footage of prey entry and exit strategies of the *Drilus* larvae, and evaluate the potential mutual evolutionary impacts. We find limited evidence for an effect of shell features and snail behavioral traits on inter- and intra-specifically differing predation rates. We also find that *Drilus* predators adjust their predation behavior based on specific shell traits of the prey. In conclusion, we suggest that, with these baseline data, this interesting predator-prey system will be available for further, detailed more evolutionary ecology studies.

## Introduction

Gastropods are among the most diverse groups of animals, and the most readily observable aspect of this diversity is in their shell form and ornamentation. Gastropod shell shape is generally considered to evolve under a strong direct influence of biotic and abiotic agents, and, at least in marine gastropods, predator-prey interactions are paramount among these [1]–[2]. On land, however, the influence of predators on snail shell evolution is less clear [3].

**Figure 1:**
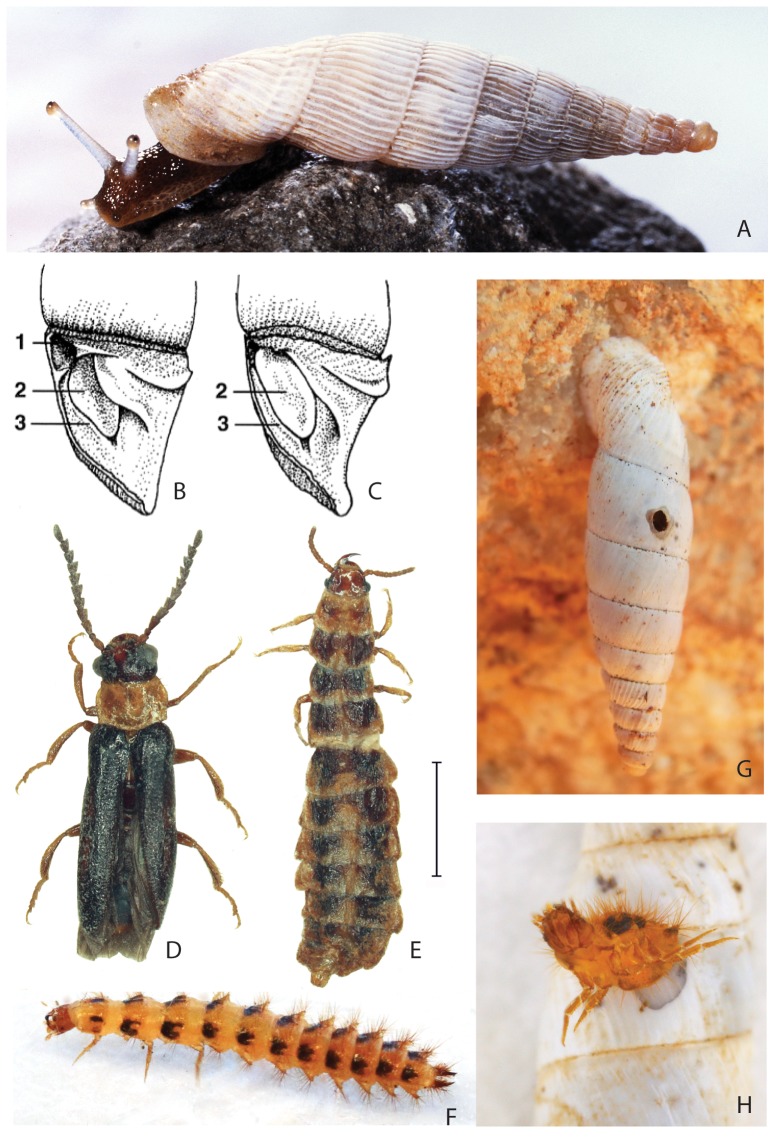
Greek *Albinaria* snails and their *Drilus* predators. A, *Albinaria hippolyti* from Crete (photo: V. Wiese). B and C, the clausilium, shown in the shell aperture after removal of the left lateral shell wall (B shows a less-obstructing, N-type clausilium, C shows a more obstructing, G-type clausilium). D and E, a male and a female, respectively, of a yet undescribed *Drilus* species from Crete (scale: 2 mm). F, a full-grown larva of *Drilus* “L” from the Peloponnese (same scale as D and E). G, an estivating *A. discolor* from the Peloponnese, with a *Drilus* exit bore hole. H, a *Drilus* “L” exiting from its prey, an *A. menelaus* from the Peloponnese.

One particularly diverse group of land snails is the clausiliid genus *Albinaria*, of which the >100 species occur abundantly on limestone rocks throughout Greece and surrounding regions (fig. 1A) [4], with most species occupying small, usually non-overlapping ranges [5]–[7]. The animals actively forage on micro-flora on the rocks during the wet months (roughly October to April), but estivate, usually with their apertures firmly sealed to the rock, and often in dense clusters, during the dry part of the year. Species differentiation is most apparent in various shell traits, such as radial ribbing, apertural folds and lamellae, and the structure of the clausilium (a door-like aperture closing apparatus and a Clausiliidae synapomorphy; figs. 1B, C).

Although *Albinaria* snails are preyed upon by a wide range of predators, including rodents, molluscivorous snails, and carabid beetles [8], their chief enemies appear to be larvae of the elaterid beetle genus *Drilus* (fig. 1D–F). These enter *Albinaria* snails by boring a hole through the shell wall (fig. 1G), killing and eating the snail's soft body, and, after molting or pupating, leaving via a second bore-hole (fig. 1H) [9]–[10]; but see below. Often, the entry and exit holes can be distinguished by their shape [8]. In many locations, more than 50% of the empty shells carry such *Drilus* bore-holes [11].

Given their intimate relationship with *Albinaria* and their shells, we expect that *Drilus* might be an important selective agent in *Albinaria* shell evolution. Indications of non-random predation by *Drilus* already exist, since Mesher and Welter-Schultes [11] found that on the island of Día (off Crete), *Drilus* attack was high in the three native *Albinaria* species, but low in a fourth, possibly introduced, species. To assess such possibilities further, we have investigated in more detail the interaction between *Albinaria* and *Drilus* in various regions in Greece.

## Biogeography and Diversity of *Drilus* Predators

**Figure 2:**
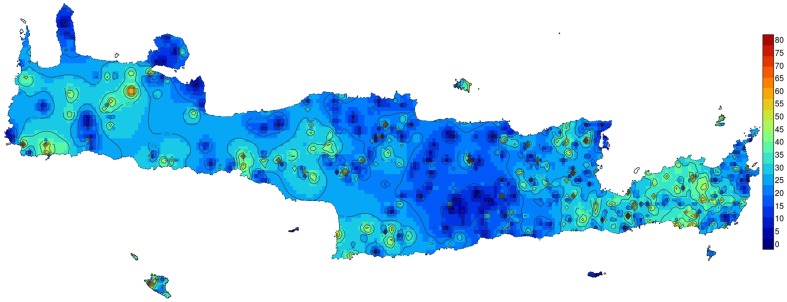
Map of Crete and surrounding islands, showing contours of regionally varying *Drilus* predation rates (given as percentages attacked shells per sample) in *Albinaria*, derived from bore-hole frequencies in 1,160 museum samples from Naturalis Biodiversity Center, Leiden, The Netherlands, Haus der Natur, Cismar, Germany, and Natural History Museum, Budapest, Hungary. Maps were drawn using inverse kriging distance calculation in R v.2.15.2 [23], with packages gstat [24], maptools (R v.0.8-27), rgdal (R v.0.8-12) and rgeos (R v.0.3-2).

We first investigated the geographic variation in predation rate, by mapping the proportions, relative to the total number of empty shells, of shells with *Drilus* bore-holes, which have a characteristic size and shape, and are easily distinguished from other types of shell damage—see fig. S1 and [8]—throughout Crete. We did this by measuring the proportion of shells with *Drilus* holes in each of 1,160 museum samples. (With “sample” we mean a number of empty shells from a single location, usually taken in a 50×50 m area; see Data files S1 and S3.) These show a pattern of high, but regionally varying predation rates, with an average of 0.239±0.168 (fig. 2). In the Peloponnese and Kephalonia, where our geographic coverage was much less complete, we found predation rates that appeared lower than in Crete, but still substantial: average  =  0.082±0.079 (n = 18 samples, 7,450 shells in total; in view of the mixed character of the samples, we refrained from testing for significance in the Crete vs. Peloponnese+Kephallonia difference). *Drilus* predation therefore accounts for a large proportion of total adult mortality [12].

**Figure 3:**
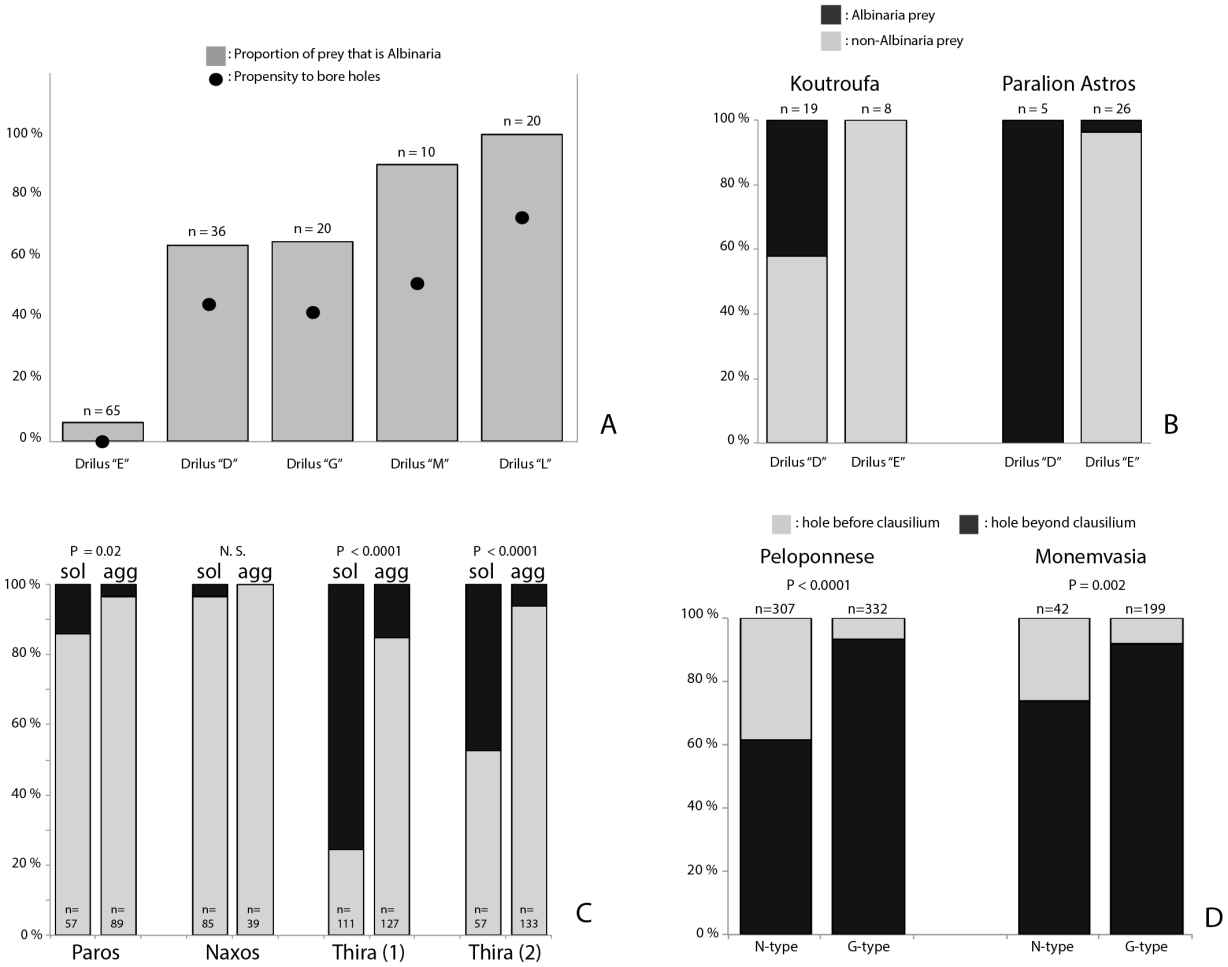
A, five *Drilus* species from the Peloponnese have different specificities for *Albinaria* as prey, and concomitant propensities to bore holes in the shell (calculated as the number of bore holes divided by the total number of prey). B, difference (*P*<0.05, Fisher's exact test) in prey specificity between *Drilus* “D” (more *Albinaria*-specific) and “E” (less *Albinaria* specific) in two localities where both species occur syntopically (these data are not included in fig. 3A). C, differences in *Drilus* predation rate (dark portion of the bar) between solitarily (“sol”) and group-wise (“agg”) estivating snails of *A. caerulea* in four 5 m^2^ plots in the islands of Paros, Naxos, and Thira (voucher numbers RMNH.MOL.84354-84363, RMNH.MOL.85192, and RMNH.MOL.85193). An aggregate was defined as a cluster of >20 snails, with distances of <2 cm separating them. A snail was considered solitary if it was >20 cm distance from a conspecific (significance tested with Chi-square test). D, positions of entrance holes in shells of species with an N-type clausilium compared with those in shells of species with a G-type clausilium, shown for the entire eastern Peloponnese as well as for the site Monemvasia, where both clausilium types occur microsympatrically. (*P*-values are derived from Fisher's exact test.)

We then used a combination of larval and adult characters, and mtDNA sequencing, to determine the number of *Drilus* species in the region, as well as their prey specificity (see Data file S2). We found that at least nine species can be distinguished (for the taxonomic and phylogenetic details we refer to Ref. [13], and Kundrata et al. unpublished manuscript; we provide a preliminary map of the species' distributions in fig. S2). For the four species that occur in Crete, we did not assess prey specificity, but for the remaining five, occurring in the Peloponnese, Zakynthos, and Kephalonia, we were able to do this by investigating bore holes and exuviae in shells of the entire snail fauna in 39 locations. The results (Fig. 3A) show that *Drilus* includes obligate *Albinaria*-specialists (e.g., *Drilus* “L”) as well as generalists that only rarely attack *Albinaria* (e.g., *Drilus* “E”). Although these results may be somewhat confounded by differences in the snail species abundance distributions, the data from two locations, Paralíon Ástros and Koutróufa, where two *Drilus* species (*D.* “D” and *D.* “E”) occur syntopically, confirm the differences in prey specificity between these two species (Fig. 3B). As in other members of the genus, our *Drilus* species tend to have small geographic ranges (e.g., *Drilus* “D,” “L,” and “M” may have range lengths of 50 km or less), which may be explained by the fact that *Drilus* females are wingless (fig. 1E) [14].

## Behavior of *Drilus* preying on *Albinaria*

Unexpectedly, we found that *Drilus* may successfully attack *Albinaria* without leaving any bore-holes in the shell. We opened 645 dead *Albinaria* shells from the Peloponnese, and out of 169 shells that had been preyed by *Drilus* (judged by the presence of exuviae or a live larva), 60 contained exuviae but showed no trace of a bore-hole. This means not only that predation rates calculated from bore-hole frequencies are underestimates, but also that *Drilus* employs more than one attack strategy.

**Figure 4:**
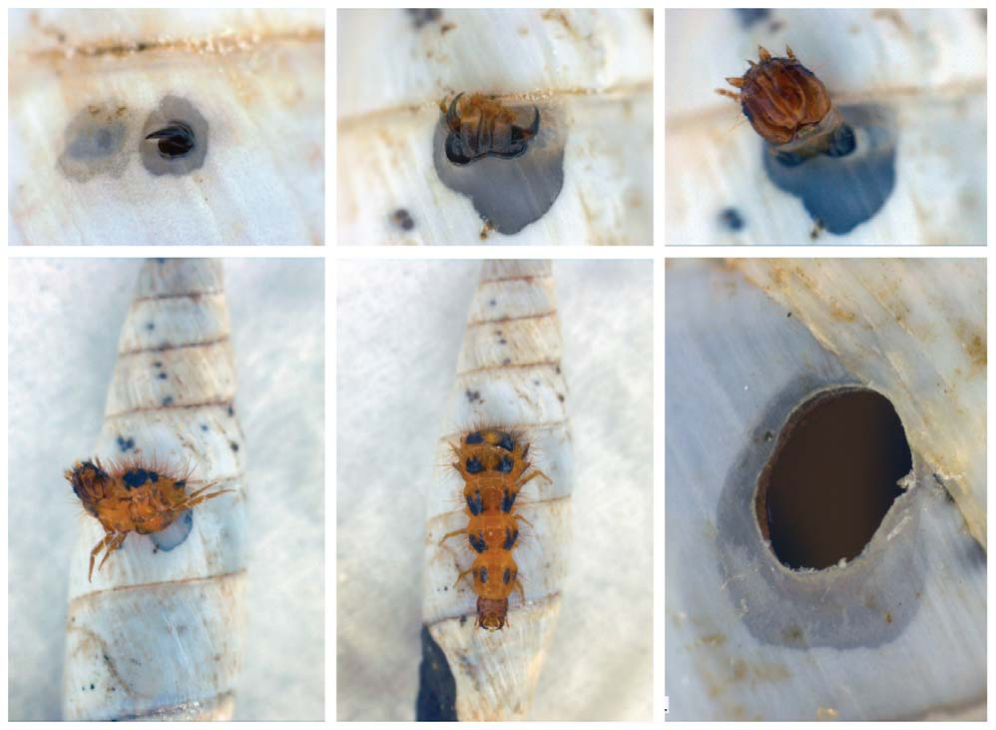
Still from Video S1, showing a *Drilus* “L” larva (in the lab) creating an exit bore-hole from within a prey *Albinaria meleaus*, followed by the lengthy procedure by which the larva emerges from the shell.

To understand better the predatory behavior of *Drilus*, we obtained three live pseudopupae (an immobile resting stage) from field-collected *Albinaria* shells, as well as live *Albinaria*, and used these for observations in the laboratory. Of these three, one remained in pseudopupal state. The other two (both *Drilus* “L”) molted into active larvae. One (obtained from an *A. edmundi*) entered an *Albinaria edmundi* via the aperture and killed it. It remained inside for 28 days, bored a hole in the shell wall to exit, but then died while emerging. However, the third one (obtained from an *A. discolor*) stayed alive and active for almost two years, and during that time consumed eight adult prey individuals. In all eight predation events (which took place under dark conditions in a box with several loose *Albinaria* individuals, i.e., not adhering to any substrate), the larva entered the snail via the aperture (after having inspected several potential prey), never boring a hole in the shell wall nor into the clausilium (Video S2). After entering a snail, it apparently attacked and ate (part of) the snail immediately, because it moved fragments of dried, undigested snail tissue outside of the aperture within three days. In total, it would remain in a shell for 22–32 days, except for one very lengthy stay inside a prey shell that lasted from September 19^th^ until May 1^st^, and possibly indicated hibernation. Each time the larva exited from an empty prey shell, it left behind an exuvia, meaning that the number of larval stages may be much larger than the three to four that had been suspected previously [15]–[16]. In all cases except one, the larva did bore a hole from within the prey shell to exit. Boring was done with the jaws and a copious amount of (possibly acidic) saliva and, based on the one occasion when it was observed from start to finish (Video S1; fig. 4) took seven hours.

These observations confirm that a *Drilus* may enter an *Albinaria* prey either by apertural-entry or by shell-boring (see also Video S2 for these behaviors in Cretan species). Similarly, it may also exit the (empty) prey shell either via the aperture or via a new bore-hole. As in other snail-predator interactions [16]–[18], each of these entry and exit strategies have their advantages and disadvantages. For example, apertural entry as well as exit may be hindered by the clausilium and/or by apertural folds that project into the lumen of the whorl. On the other hand, it may be less costly than boring in terms of time, energy, and saliva (if the saliva indeed helps dissolve the shell). Entering and perhaps also exiting the shell by boring may be hindered by the presence of dense and/or tall radial ribs on the shell surface, but is more costly. Finally, it should be noted that shell features that impede predator entry will primarily impose a selection pressure on the prey (by ensuring its survival), whereas those that impede predator exit will primarily select for predator traits (by causing the predator to die within the prey shell).

## Potential Evolutionary Impact of Predator-Prey Interaction on the Prey

We may predict that more heavily-ribbed *Albinaria* species [19] are better protected against boring (and therefore show a lower proportion of bored shells and/or more bore-hole failures) and that *Albinaria* species with a more completely obstructed aperture [20] are better protected against apertural-entry (and therefore show more bored shells). As a preliminary test of these predictions, we scored numbers of bored and intact shells in pairs of syntopic *Albinaria* species, exposed to the same *Drilus* species, but differing in either radial rib height or apertural obstructions. Our results (Table S1) do not provide consistent support for the predictions. At Argínia, the strongly ribbed *A. adrianae* has, as predicted, a lower proportion of bored shells than the smooth *A. contaminata*, but at Póros, no difference was found. Bore-hole failures do not differ among the four populations. At one of the three sites where a species with a partly-obstructed aperture (an N-type clausilium) co-occurs with a species with a completely obstructed (G-type) aperture, the latter has fewer bored shells, while at the other two locations, no difference was found. Obviously, these results are inconclusive since they are based on small numbers of populations and should be repeated with a larger-scale study, including more species and more locations.

We also obtained some data that suggest that predation by *Drilus* may be non-random within a single *Albinaria* population and hence may cause natural selection on variable traits. In a population of *A. krueperi*, preyed upon by *Drilus* “G”, we found that predation (derived from exuviae in the shells) was predominantly towards the smaller individuals; however, such a response was not found in a population of *A. adriani*, preyed upon by *Drilus* “D” and “E” (Table S2). In addition, in a population of *A. caerulea* from the Cyclades, we found that predation risk (derived from bore-hole data) was greater in snails estivating in isolation than in snails estivating in clusters (fig. 3C), which may be one way in which such clustered estivation (a conspicuous behavioral pattern in most *Albinaria* species) evolved.

## Potential Evolutionary Impact of Predator-Prey Interaction on the Predator

In contrast to the evolutionary impact that the predator may have on the prey, the reverse may also be the case. *Albinaria* snails are, at least during the dry months of the year, often firmly sealed against the substrate, whereas most of the other species that we recorded as *Drilus* prey tend to estivate in the soil. We may therefore expect that *Drilus* species that are more specialized in feeding on *Albinaria*, have a greater tendency to bore holes for entering, and, probably more importantly, for exiting the shell (an inability to do the latter would mean death for the *Drilus* larva). Indeed, we find a positive correlation between *Albinaria*-specificity and hole-boring tendency among the five *Drilus* species from the Peloponnese and Kephalonia (fig. 3A).

Finally, another indication that the prey shell morphology affects the predator was obtained when we compared the positions of entrance holes in shells of species with an N-type clausilium (*A. argynnis*, *A. discolor*, and *A. solicola*; all from the Eastern Peloponnese) with those in shells of species with a G-type clausilium (*A. adriani*, *A. edmundi*, and *A. campylauchen*, all roughly sympatric with the previous three). We found that in the N-type group, more shells had a bore-hole in the outer shell wall between the aperture and the clausilium than in the G-type group (fig. 3D). Since the N-type species have a clausilium that does not completely close off the aperture, whereas the G-type clausilium does, these data may indicate that predator populations faced with G-type prey have evolved a shell entry strategy by which the hole is bored at a position beyond the obstruction. However, the same difference is seen in Monemvasia, where the N-type *A. discolor* lives syntopically with the G-type *A. campylauchen*. This suggests that, rather than an evolved behavioral difference between predator populations, the predator may also be able to detect the clausilium type before boring a hole and that the difference in strategy is due to a behavioral response, rather than an evolved, fixed behavior (fig. 3D).

## Conclusion

Given the complexity of this predator-prey system, with multiple predator species, multiple prey species, varying prey specificity, and complex small-scale biogeographic patterns, we will not hazard a conclusion on the impact of these interactions on shell evolution in *Albinaria*. However, we think we have shown that detailed study of the natural history of these ecological interactions reveal a potential for such evolutionary responses. Elements from our study may provide starting points for further work, specifically targeted at understanding the possible predator-prey arms races or patterns of escalation in this system, an approach that has proved successful in a similar terrestrial system consisting of tropical micro-snails and shell-boring slugs [21]–[22].

## Additional Methods Details

Sampling of live specimens was done at the locations with the following coordinates: 37°09.694′ N, 22°48.745′ E and 37°20.132′ N, 22°45.769′ E. No specific permissions were required for the accomplished field sampling activities by the University of Patras. The field studies were conducted exclusively in public and not protected land areas. The field studies did not involve any endangered or protected species. Sampling was conducted according to the main legal texts in the field of biodiversity (UN Convention on Biodiversity), which became part of the Greek legislation in 1994 (law 2204), and the EU Directive 2004/35 regulating environmental liability. Animal capturing, handling and killing was designed to avoid distress and unnecessary suffering to the animals as laid down in the Council Directive 86/609/EEC, art. 7.4 and the Council of Europe (CoE) European Convention for the protection of vertebrate animals used for experimental and other scientific purposes (1986, ETS 123). All relevant information was also given to the Bioethics Committee of the University of Patras".

## Supporting Information

Figure S1 Fig. S1. *Drilus* bore hole proportions (both entry and exit holes), based on measurements taken from shells of *A. discolor* and *A. adriani* from Agios Andreas and in *A. discolor, A. campylauchen* and *A. discolor x A. campylauchen* hybrids from Monemvasia.
(PDF)

Figure S2 Distribution map for *Drilus* morphospecies in the Peloponnese and surrounding area. In addition to morphospecies “D,” “E,” “G,” “L,” and “M,” mentioned in the text, a location for a sixth species, “U,” is also shown. Each dot represents one or more specimens. Bicolor dots indicate syntopic occurrence of multiple species. Scale bar  =  100 km.
(PDF)

Data File S1 Data Collection Codes: This file contains detailed data on the snail samples, containing *Drilus* larvae, adults, or exuviae, collected in Greece for this study, as well as on the field localities. Data available from the Dryad Digital Repository:.
(XLSX)

Data File S2 Data Voucher Numbers: This file contains detailed information on all *Drilus* specimens used for this study. Data available from the Dryad Digital Repository:.
(XLSX)

Data File S3 Data Predation Crete: This file contains data used to assess geographic distribution of *Drilus* predation rates in *Albinaria* prey across Crete. Material derived from Naturalis Biodiversity Center, Leiden, and Haus der Natur, Cismar. Data available from the Dryad Digital Repository:.
(XLS)

Table S1 *Drilus* predation in co-occurring prey species pairs differing in shell traits. Significance was tested with Fisher's exact test.
(DOCX)

Table S2 Five shell traits in *Albinaria krueperi* and *A. adriani* populations, with indications for selection by *Drilus* predation. Shell height and width were measured with hand-held calipers, the other traits with a graded ocular. *Drilus* attack was ascertained by the presence of a bore hole and/or an exuvia in the shell. Significance was tested with 2-sample t-test in R.
(DOCX)

Video S1 A *Drilus* “L” larva (in the lab) creating an exit bore-hole from within a prey *Albinaria meleaus*, followed by stills from the lengthy procedure by which the larva emerges from the shell.
(WMV)

Video S2 Series of three clips (footage sped up 64x), taken in the lab: First, *Drilus longulus* larva entering an *Albinaria* (*cretensis* complex) shell via an entry bore-hole, followed by footage in which entry is made by a *Drilus* sp. via the aperture of an *Albinaria* (*cretensis* complex) snail. Finally, a *Drilus* “L” larva selecting and entering an *A. menelaus* snail via the aperture.
(WMV)
