# Supplementary material for: Predator-Prey Interactions between Shell-Boring Beetle Larvae and Rock-Dwelling Land Snails
Source: PLoS One. 2014 Jun 25;9(6):e100366. doi: 10.1371/journal.pone.0100366 (PMC4070943; doi:10.1371/journal.pone.0100366)
Supplement: Figure S1 — Fig. S1. Drilus bore hole proportions (both entry and exit holes), based on measurements taken from shells of A. discolor and A. adriani from Agios Andreas and in A. discolor, A. campylauchen and A. discolor x A. campylauchen hybrids from Monemvasia. (PDF) [file pone.0100366.s001.pdf]

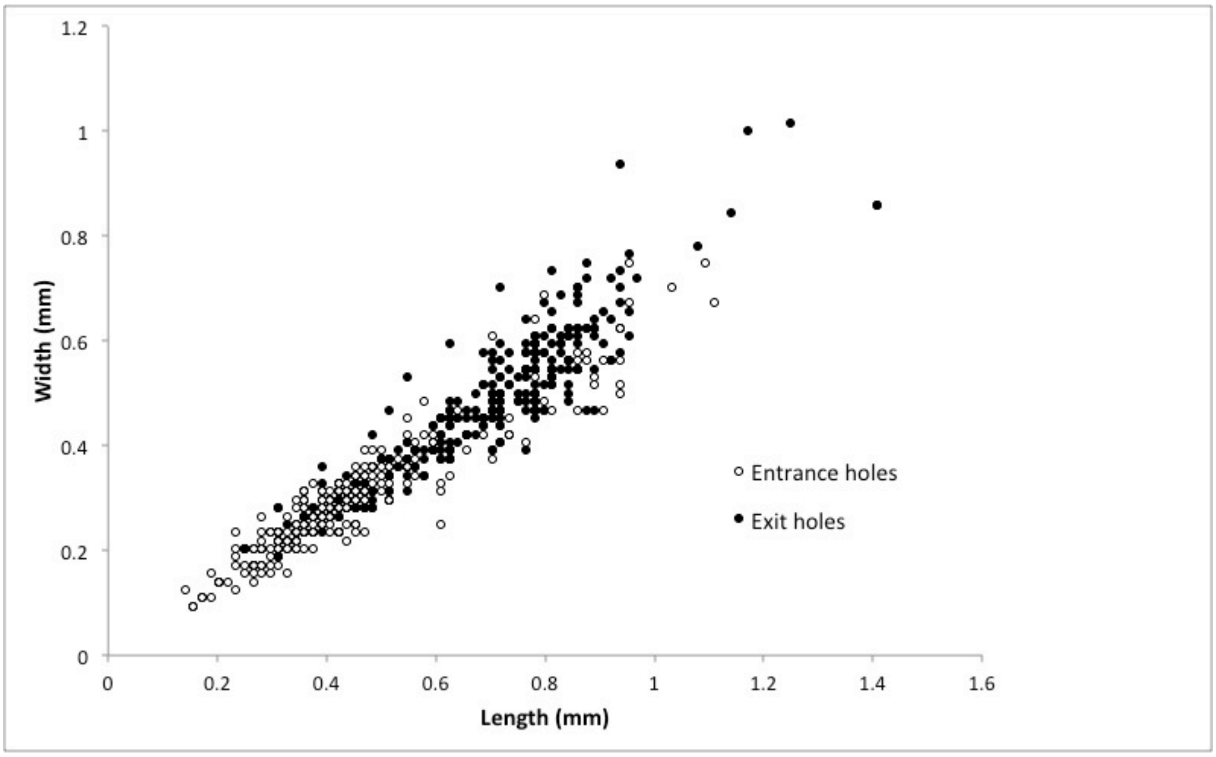

Fig. S1. *Drilus* bore hole proportions (both entry and exit holes), based on measurements taken from shells of *A. discolor* and *A. adriani* from Agios Andreas and in *A. discolor, A. campylauchen* and *A. discolor x A. campylauchen* hybrids from Monemvasia.
