# Supplementary material for: Predator-Prey Interactions between Shell-Boring Beetle Larvae and Rock-Dwelling Land Snails
Source: PLoS One. 2014 Jun 25;9(6):e100366. doi: 10.1371/journal.pone.0100366 (PMC4070943; doi:10.1371/journal.pone.0100366)
Supplement: Figure S2 — Distribution map for Drilus morphospecies in the Peloponnese and surrounding area. In addition to morphospecies “D,” “E,” “G,” “L,” and “M,” mentioned in the text, a location for a sixth species, “U,” is also shown. Each dot represents one or more specimens. Bicolor dots indicate syntopic occurrence of multiple species. Scale bar = 100 km. (PDF) [file pone.0100366.s002.pdf]

Distribution of morphospecies in the Peloponnese and Ionian Islands

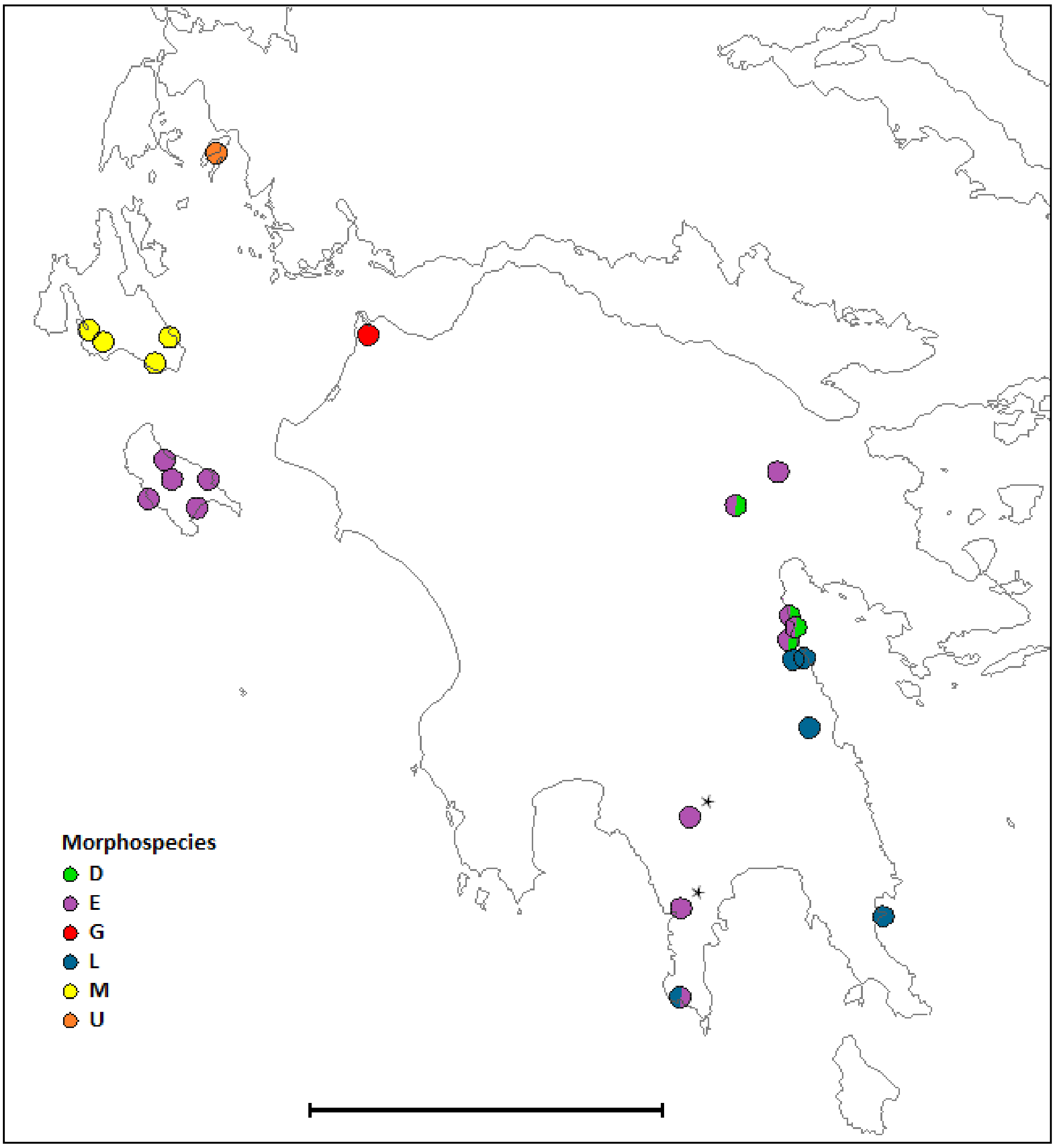

Figure S2. Distribution map for *Drilus* morphospecies in the Peloponnese and surrounding area. In addition to morphospecies “D,” “E,” “G,” “L,” and “M,” mentioned in the text, a location for a sixth species, “U,” is also shown. Each dot represents one or more specimens. Bicolor dots indicate syntopic occurrence of multiple species. Scale bar = 100 km.
