# Supplementary material for: Predator-Prey Interactions between Shell-Boring Beetle Larvae and Rock-Dwelling Land Snails
Source: PLoS One. 2014 Jun 25;9(6):e100366. doi: 10.1371/journal.pone.0100366 (PMC4070943; doi:10.1371/journal.pone.0100366)
Supplement: Table S1 — Drilus predation in co-occurring prey species pairs differing in shell traits. Significance was tested with Fisher's exact test. (DOCX) [file pone.0100366.s006.docx]

**Table S1:** *Drilus* predation in co-occurring prey species pairs differing in shell traits. Significance was tested with Fisher’s exact test.

| locality | *Albinaria* species | trait | *Drilus* species | sample size (n) | shells with bore hole(s) | prop. failed bore holes | prop. of bored shells | *P* |
| --- | --- | --- | --- | --- | --- | --- | --- | --- |
| Poros | *A. contaminata* | smooth | “M” | 168 | 3 | 2/5 | 0.018 | N.S. |
| Poros | *A. adrianae* | ribbed | “M” | 133 | 6 | 2/11 | 0.045 |  |
| Arginia | *A. contaminata* | smooth | “M” | 285 | 27 | 5/38 | 0.094 | 0.008 |
| Arginia | *A. adrianae* | ribbed | “M” | 233 | 8 | 0/9 | 0.034 |  |
| Agios Andreas | *A. adriani* | obstructed aperture | “L” | 40 | 4 | n/a | 0.100 | 0.036 |
| Agios Andreas | *A. discolor* | less obstructed aperture | “L” | 754 | 187 | n/a | 0.248 |  |
| Leonidio | *A. edmundi* | obstructed aperture | “L” | 623 | 81 | n/a | 0.130 | N.S. |
| Leonidio | *A. argynnis* | less obstructed aperture | “L” | 60 | 7 | n/a | 0.117 |  |
| Monemvasia | *A. campylauchen* | obstructed aperture | “L” | 1007 | 208 | n/a | 0.207 | N.S. |
| Monemvasia | *A. discolor* | less obstructed aperture | “L” | 151 | 36 | n/a | 0.238 |  |
