# Supplementary material for: Predator-Prey Interactions between Shell-Boring Beetle Larvae and Rock-Dwelling Land Snails
Source: PLoS One. 2014 Jun 25;9(6):e100366. doi: 10.1371/journal.pone.0100366 (PMC4070943; doi:10.1371/journal.pone.0100366)
Supplement: Table S2 — Five shell traits in Albinaria krueperi and A. adriani populations, with indications for selection by Drilus predation. Shell height and width were measured with hand-held calipers, the other traits with a graded ocular. Drilus attack was ascertained by the presence of a bore hole and/or an exuvia in the shell. Significance was tested with 2-sample t-test in R. (DOCX) [file pone.0100366.s007.docx]

**Table S2:** Five shell traits in *Albinaria krueperi* and *A. adriani* populations, with indications for selection by *Drilus* predation. Shell height and width were measured with hand-held calipers, the other traits with a graded ocular. *Drilus* attack was ascertained by the presence of a bore hole and/or an exuvia in the shell. Significance was tested with 2-sample t-test in R.

| **shell trait (mm)** | ***A. k* with *Drilus*** | ***A. k* without *Drilus*** | ***P*** | ***A. a* with *Drilus*** | ***A. a* without *Drilus*** | ***P*** |
| --- | --- | --- | --- | --- | --- | --- |
| shell height | mean=11.98  s.d.=1.119  n=63 | mean=12.71  s.d.=1.205  n=135 | 0.00007 | mean=13.45  s.d.=0.900  n=67 | mean=13.58  s.d.=0.989  n=88 | N.S. |
| shell width | mean=3.04  s.d.=0.164  n=68 | mean=3.12  s.d.=0.177  n=148 | 0.00072 | mean=3.52  s.d.=0.145  n=100 | mean=3.51  s.d.=0.157  n=121 | N.S. |
| aperture height | mean=3.18  s.d.=0.234  n=65 | mean=3.33  s.d.=0.272  n=139 | 0.00017 | mean=3.50  s.d.=0.246  n=63 | mean=3.59  s.d.=0.263  n=85 | 0.03113 |
| aperture width | mean=2.53  s.d.=0.212  n=61 | mean=2.65  s.d.=0.208  n=137 | 0.00035 | mean=2.87  s.d.=0.189  n=65 | mean=2.92  s.d.=0.205  n=82 | N.S. |
| lip width | mean=0.373  s.d.=0.0867  n=60 | mean=0.416  s.d.=0.0808  n=136 | 0.00086 | mean=0.503  s.d.=0.1250  n=78 | mean=0.535  s.d.=0.1151  n=96 | N.S. |
